# Distress and its influencing factors among Chinese patients with incidental pulmonary nodules: a cross-sectional study

**DOI:** 10.1038/s41598-023-45708-w

**Published:** 2024-01-12

**Authors:** Jingmin Yuan, Fenglin Xu, Hui Ren, Mingwei Chen, Sifang Feng

**Affiliations:** 1 Health Science Center, Yangtze University, Jingzhou, China; 2 Department of Pulmonary and Critical Care Medicine, The First Affiliated Hospital of Xi’an Jiaotong University, NO.277 Yanta West Road, Xi’an, China; 3 Department of Nursing, Hubei College of Chinese Medicine, Jingzhou, China; 4 International Exchange Office, The First Affiliated Hospital of Xi’an Jiaotong Univeristy, Xi’an, China

**Keywords:** Cancer, Respiratory tract diseases

## Abstract

The study aims to investigate the distress level and its influencing factors in Chinese pulmonary nodules patients. A total of 163 outpatients in a tertiary hospital in Xi'an, China, were recruited and investigated by using the Impact of Event Scale, Decision Conflict Scale, Consultation Care Measure, Lung Cancer Worry Scale and a demographic questionnaire. The logistic regression model was used to identify the factors of distress. The mean IES score was 37.35 ± 16.65, which was a moderate level. Patients aged 50–60 years, with higher decision conflicts scores, lower physician–patient communication quality score, and who are anxious about the results of future tests or treatments had higher distress score. Distress levels were moderate in patients with pulmonary nodules. Communication between medical staff and patients is extremely important for the management of pulmonary nodules, which affects the quality of the patient's decision-making and his level of distress.

## Introduction

Lung Cancer has the second-highest incidence rate and the highest mortality rate in the world^1^, and it is the most common cancer in China with new cases and deaths accounting for nearly 40% in the whole world^2^. Early detection is important for lung cancer prevention and management. Results of the National Lung Screening Trial (NLST) showed that screening with low-dose computed tomography (LDCT) can reduce lung cancer mortality by 20%^3^. The latest study shows that one-off LDCT screening in high-risk populations in China can reduce lung cancer mortality by 30%^4^. In 2012, the Chinese government launched a key national plan for public health, Cancer Screening Program in Urban China (CANSPUC), in which participants at high risk aged 40–74 were provided free LDCT test for lung cancer screening^5^.

With the widespread use of computed tomography (CT) technology, pulmonary nodules are becoming more common. Nearly 26.3% of pulmonary nodules were found on annual routine checkups^6^. After being diagnosed, patients have questions about the meaning of the nodules.

With the increasing prevalence of non-smoking related lung cancer in Asian countries, LDCT screening for non-smoking population is widely utilized in Asian countries and regions, such as China, Taiwan (province of China), Korea, Japan. Population anxiety about lung cancer was high in China due to patients died of stage IV lung cancer, unknown risk factors for non-smoking lung cancer, and safe strategies culture in Asian population culture, especially in non-smoking and female populations^7^.

Since pulmonary nodules have the potential to become early lung cancer lesions, patients may become anxious and depressed. Harris et al. believed that this "near cancer" diagnosis would bring various harms to patients, including physical harms, psychological harms, financial strain, and opportunity costs^8^. It is worth noting that very few patients benefit from lung cancer screening (most of them are benign), but nearly all patients are at risk of different types of harms from detection and evaluation of a pulmonary nodule^3,9^.

It is difficult to make an accurate judgment of benign and malignant after the first detection of pulmonary nodules. Patients care about the next step in the diagnosis and treatment plan^10^. Common options for the management of pulmonary nodules in the guidelines include CT surveillance, biopsy, and excision. However, each option has its advantages and disadvantages^11^, and it is a challenge for doctors and patients to choose which one to use. It involves the characteristics of the nodule itself, the patient's history of exposure to lung cancer risk factors, the doctor's judgment, and the patient's personal preference. Patients may be caught in a decision-making dilemma.

Decision conflict is when individuals face choices involving risks or unknown outcomes, they need to evaluate the potential benefits and risks corresponding to the options, weigh the value of making a decision, and prepare for losing the advantages of the rejected options^12^. Decision conflict can lead to delayed decision-making, reduce treatment compliance, and affect the patient's psychology. Studies on breast cancer and diabetes patients have shown that there is a positive correlation between decision conflict and distress, and the uncertainty in decision-making can lead to individual psychological and emotional distress^13,14^. Communication between doctors and patients is very important for this kind of decision conflict faced by patients. This communication is not just the simple transmission of medical information, but also requires emotional exchange. Some researchers believe that high-quality communication can effectively alleviate the distress level of patients.

In the context of the high incidence of lung cancer, the distress level of patients with pulmonary nodules in China is unknown. Since the nature of pulmonary nodules requires the patient to further choose a certain way to determine, the patient needs to communicate with the medical staff during the decision-making process, and decisional conflict, communication quality with doctor, and lung cancer worry are closely related to the patient in this process. The purpose of this study was to investigate the distress level and its influencing factors in Chinese patients with incidental pulmonary nodules.

## Method

### Aim

The study aims to investigate the distress level and its influencing factors in Chinese pulmonary nodules patients.

### Design

This is a cross-sectional study and adhered to the STROBE checklist for observational research.

### Sample

Participants were eligible for this study if they were 18 years of age or older with a diagnosis of pulmonary nodule (with diameter less than 30 mm) after opportunistic CT examination for the first time. People who had mental illnesses or any type of cancer were excluded. Kendall et al.^15^ suggested that the study sample size should be 5–10 times of the study factors. This study contains 12 variables, considering the sample loss of 20%, the sample size of this study needs to be at least 150 participants. Using convenience sampling, 165 pulmonary patients at the respiratory clinic of a tertiary hospital in Xi’an were recruited from July 2021 to October 2021. Two patients declined to participate for time reasons, and 163 participants were eventually included in the study.

### Data collection

**Figure 1:**
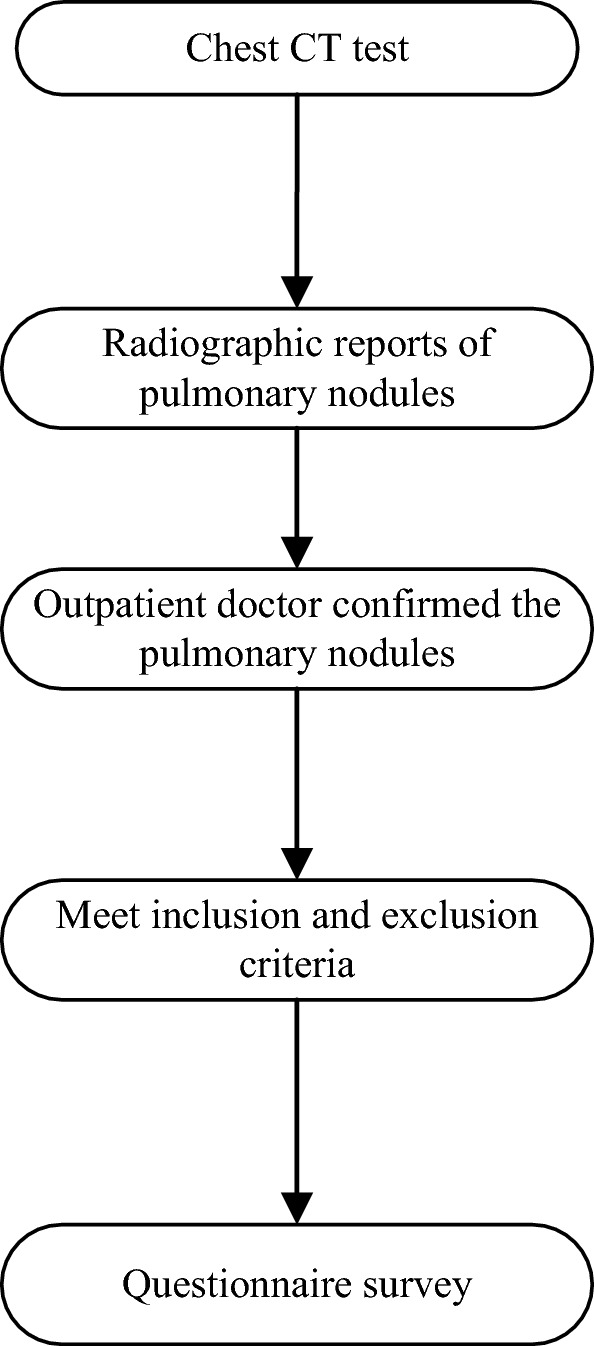
Data collection timing flow chart.

All the questionnaire and scales were completed by the patient alone. After the participants completed the questionnaire, the researchers immediately checked the questionnaire and collected it on the spot. The data collection timing flow chart see Fig. 1.

#### Social-demographic characteristics

Socio-demographic questionnaire included age, gender, education level, marital status, employment status, income level, residence, smoking history, relatives with lung cancer, if the patients worried about getting lung cancer someday, and if the patients anxious about the results of future tests/treatments.

#### Distress

Impact of Event Scale (IES) was used to measure distress. IES, originally designed by Horowitz in 1979, measures an individual's current level of subjective distress due to the impact of a specific event. In 2003, Zhao et al. translated it into a Chinese version^16^. The scale contains 15 items and 2 dimensions, including intrusion (7 items) and avoidance (8 items). Each item was scored on a 4-point scale (0 = not at all, 1 = rarely, 3 = sometimes, and 5 = often). The total scale score ranges from 0 to 75^17^, with higher scores indicating a greater frequency of intrusive thoughts and attempts at avoidance. In this study, according to other research, a total score of 0–25 was classified as mild or below level, and above 26 was considered moderate or high level^18^.

#### Decision conflict

The Decision Conflict Scale (DCS) was used to measure the decision conflict of patients with pulmonary nodules when deciding the next treatment or surveillance plan. DCS was originally designed by O’Connor in ^12^ and was later translated into a Chinese version by Li et al.^19^ The scale contains 16 items divided into 3 subscales: including information and values, decision support and effectiveness and decision uncertainty. The scale was scored using the Likert 5-level scoring method, and the initial score was converted into 0–100 points to obtain the total score.

#### Communication quality with doctor

Consultation Care Measure (CCM) is used to measure the quality of communication between doctors and patients about pulmonary nodules. The CCM is designed based on a patient-centered communication model^20,21^ to measure patients' perceptions of communication quality after communicating with doctors. The primary measure of the perception of communication was the statement, “The overall quality of communication with your provider (who is caring for your nodule) is excellent,” rated on a 7-point Likert scale from “very strongly disagree” to “very strongly disagree” strongly agree.”^18^. Higher scores indicate that patients perceive the higher quality of communication.

#### Lung cancer worry

The Lung cancer worry scale (LCWS)was used to measure fear and anxiety in patients with pulmonary nodules about being diagnosed with lung cancer in the future. The scale was modified from the cancer worry scale by changing breast cancer to lung cancer and by changing mammograms to future tests/treatments^22^. Clark et al. applied it for the first time in patients with pulmonary nodules, and there were four questions in the scale. Since we included patients diagnosed for the first time, only two questions were included: “How worried are you about getting lung cancer someday?” and “What is your current anxiety level about the results of future tests/treatments?" These two questions were dichotomized as "not worried" vs. "worried" and "not anxious" vs. "anxious".

### Ethics and consent statement

Our research was approved by the Medical Ethics Review Committee of the First Affiliated Hospital of Xian Jiaotong University, and all methods were performed in accordance with the relevant guidelines and regulations. Patients were informed about the study and invited to participate the survey. The data were anonymous, and coded using participant-created codes.

### Data analyses

IBM SPSS Statistics software version 26.0 was used to analyse data. The demographic data were summarized as the means, standard deviations, and ranges for continuous variables and as frequency counts (percentages) for categorical variables. We calculated the means and standard deviations of distress and 2 dimensions, and reported low or mild and moderate or high levels using frequencies and percentages. The differences in distress according to demographic characteristics were analysed using t-tests and one-way analysis of variance (ANOVA). Pearson's correlation analyses were used to investigate the correlations between distress and both decision conflict and communication quality score. Multivariate logistic regression model was constructed to explore the influencing factors of distress among pulmonary nodules patients. All socio-demographic factors, decision conflict, communication quality, lung cancer worry were included in the multilevel analysis. An alpha level of 0.05 was used for the assessment of statistical significance.

### Validity and reliability

All manually entered data were double-checked. The Cronbach’s α of IES, the intrusion and avoidance subscale are 0.90, 0.88 and 0.81, respectively^16^. DCS has good reliability and validity, with Cronbach’s α coefficient of 0.897 and content validity index of 0.950^23^. Cronbach’s α for LCWS used in relation to breast and prostate cancer ranges from 0.71 to 0.86.

## Results

### Demographic characteristics

A total of 163 patients with incidental pulmonary nodules were included in this study, with an average age of 54 years (SD = 12.9, range 27–85), of whom 91 (55.8%) were female. Most participants were married (93.9%). Nearly half (47.2%) of patients with pulmonary nodules were worried about being diagnosed with lung cancer in the future, and about 33.7% were worried about the results of their next test. Detailed demographic characteristics are shown in Table 1.

**Table 1:** Summary of the associations between demographic and distress (n = 163).

Characteristic	n	Distress Mean ± SD	*t/F*	*p*
Age			2.026	0.135
18–49	52	33.5 ± 18.9		
50–60	69	39.1 ± 15.8		
≥ 60	42	39.1 ± 14.5		
Gender			− 0.623	0.534
Female	91	36.6 ± 17.6		
Male	72	38.3 ± 15.4		
Educational level			1.354	0.261
Primary school and below	39	38.6 ± 12.6		
Middle/high/technical secondary school	78	38.7 ± 18.7		
College and above	46	33.9 ± 15.7		
Marital status			0.846	0.416
Married	153	37.5 ± 16.9		
Unmarried and others	10	33.9 ± 13.1		
Employment status			0.958	0.341
Employed	113	38.3 ± 15.3		
Unemployed/retired/other	50	35.3 ± 19.3		
Income (RMB per person per month,equivalent to US$)			1.078	0.343
≤ 4000 $580)	69	35.6 ± 19.6		
4001–5000 ($581–725)	40	40.5 ± 13.9		
> 5000 ($725)	54	37.2 ± 14.2		
Residence			2.188	0.031*
Urban	108	39.3 ± 16.8		
Urban to rural/Rural	55	33.5 ± 15.8		
History of smoking			0.393	0.696
No	130	37.6 ± 15.9		
Yes	33	36.2 ± 19.2		
First degree relative with lung cancer			− 4.177	0.000**
No	145	35.6 ± 16.1		
Yes	18	51.2 ± 14.8		
Lung Cancer Worry Scale1: How worried are you about getting lung cancer someday?			− 3.815	0.000**
Not worried	86	32.8 ± 17.4		
Worried	77	42.4 ± 14.3		
Lung Cancer Worry Scale2: What is your current anxiety level about the results of future tests/treatments?			2.904	0.004**
Not anxious	108	34.8 ± 16.9		
Anxious	55	42.3 ± 15.1		

*p < .05. **p < .01.

### Distress, decision conflict, and communication quality

The mean and standard deviation of distress, decision conflict, and communication quality scores were 37.6 (SD = 16.7), 51.32 (SD = 19.5), and 3.3 (SD = 1.7), respectively.

About 73.6% (n = 120) of patients reported a moderate or high level of distress. (Table 2).

**Table 2:** Distress among incidental pulmonary nodules patients scale scores (n = 163).

Variables	Scale level Mean ± SD	Item level Mean ± SD	N (%)	
			Low/mild level	Moderate/high level
Distress	37.4 ± 16.6	2.5 ± 1.1	43 (26.3)	120 (73.6)
Intrusion	17.9 ± 8.4	2.5 ± 1.2	–	–
Avoidance	19.3 ± 9.0	2.4 ± 1.1	–	–

### Relationships among distress, demographic characteristics, decision conflict, lung cancer worry, and communication quality

Table 1 shows the relationship between distress and demographic characteristics and lung cancer worry. The correlation analyses showed that decision conflict was positive associated with distress (*r* = 0.597, *p* < 0.001) while communication quality was negatively associated with distress (*r* = 0.403, *p* < 0.001).

### Influencing factors of distress among incidental pulmonary nodules patients

The logistic regression results showed that decision conflict, age, communication quality, and anxiety level about future test results were the main factors that influenced distress among incidental pulmonary nodules patients. (Table 3).

**Table 3:** Factors influencing distress among patients with incidental pulmonary nodules.

Variables	B	OR	95% CI	*p*
Intercept	− 9.312	0		
Decision conflict	0.112	1.118	(1.068, 1.187)	0.000**
Gender				
Female		1	Reference	
Male	1.218	3.381	(0.779, 18.277)	0.123
Age				
18–49		1	Reference	
50–60	1.606	4.984	(1.17, 25.392)	0.038*
≥ 60	0.746	2.109	(0.255, 21.249)	0.498
Educational level				
Primary school and below		1	Reference	
Middle/High/technical secondary school	0.485	1.624	(0.187, 17.374)	0.670
College and above	− 1.339	0.262	(0.016, 3.691)	0.325
Marital status				
Married		1	Reference	
Unmarried and others	1.575	4.832	(0.438, 83.365)	0.234
Income				
≤ 4000 ($580)		1	Reference	
4001–5000 ($581–725)	0.263	1.301	(0.246, 7.412)	0.758
> 5000 ($725)	2.131	8.426	(1.164, 91.808)	0.053
Residence				
Urban		1	Reference	
Urban to rural/Rural	0.521	1.684	(0.358, 8.778)	0.516
History of smoking				
No		1	Reference	
Yes	− 1.214	0.297	(0.04, 2.009)	0.217
First degree relative with lung cancer				
No		1	Reference	
Yes	2.079	7.997	(0.476, 407.07)	0.215
Communication quality score	− 0.705	0.494	(0.291, 0.777)	0.004**
Lung Cancer Worry Scale1: How worried are you about getting lung cancer someday?				
Not worried		1	Reference	
Worried	0.909	2.482	(0.694, 9.696)	0.171
Lung Cancer Worry Scale2: What is your current anxiety level about the results of future tests/treatments?				
Not anxious		1	Reference	
Anxious	2.602	13.484	(2.633, 98.982)	0.004**

*p < .05. **p < .01.

## Discussion

### Distress level of pulmonary nodule patients

This study investigated the distress level and its influencing factors in Chinese patients with incidental pulmonary nodules. It was found that the vast majority of patients developed a certain level of distress after being diagnosed with a pulmonary nodule. This result is similar to that of Moseson and Slatore, who used IES to investigate patients with pulmonary nodules^18,24^.

Many patients with pulmonary nodules have certain anxiety and distress emotions^25^. Although only about 4% of pulmonary nodules will eventually be diagnosed as lung cancer^3^, it is difficult to make an accurate judgment immediately^26^, so the “near-cancer” diagnosis is a big burden to patients. Some patients even thought they had cancer immediately after being diagnosed with a pulmonary nodule^27^.

Horowitz described the connotations of the 2 dimensions of event impact. Intrusions are thoughts and images that spontaneously, involuntarily appear in the mind, with troubled dreams, strong pangs, and waves of feelings. Avoidance responses include ideational constriction, denial of the meaning and outcome of events, numbness of sensations, inhibition of behavior, or counterphobic activity ^28^. This study showed that patients with pulmonary nodules have moderate scores in both dimensions, suggesting that the two types of thoughts exist in patients. When a patient is diagnosed with a pulmonary nodule, there is a spot in the lung, which reminds the patient from time to time that he has a pulmonary nodule and it has the potential to become lung cancer. However, since most pulmonary nodules require regular follow-up, under the advice of doctors, patients will take the initiative to suggest to themselves and try to avoid thinking about it.

### Influencing factors of distress

Decision conflict is an important influencing factor of patients' distress. Pulmonary nodules are poorly understood by patients^27^, and their decisions require a wealth of information from their doctors, as well as an understanding of the pros and cons of each option. However, doctors give patients less information due to several factors. This may be related to the fact that pulmonary nodules are very common, doctors need to deal with a large number of patients, and their time and energy are limited^26^. In addition, physicians may be worried that giving too much information would be overwhelming and might actually increase patient distress, so they seldom directly inform the risk of lung cancer and other details about surveillance to their patients^29^. Based on the current context, the British Thoracic Society guidelines suggest that the involvement of lung cancer nurses in further communication with patients would be beneficial^30^. However, these nurses need to be skilled in the prevention and management of lung cancer and pulmonary nodules, such as epidemiology, risk factors, screening methods, and further management of pulmonary nodules (including advantages and disadvantages). In this process, the idea of shared decision-making can be applied. In addition, since in-depth conversation with patients may involve patients in decision-making, nurses need to receive specialized training to provide decision-making Support for patients, such as The Ottawa Decision Support Tutorial^31^.

The communication quality score was also entered into the regression model. It is similar to the results of Slatore et al., the higher the quality of patient self-rated communication, the lower the distress level^24^. In the current clinical decision-making, the paternalistic or informed model is mostly used, which has problems such as low degree of information sharing and low decision satisfaction^23^. A core criterion for high-quality decision-making is whether the choice is in line with the patient's values, goals, and preferences. This requires medical staff to learn more about patients' thoughts and consider personal preferences during the communication process. In addition, the communication between doctors and patients about the decision-making of the next diagnosis and treatment plan is not only a one-way information transfer of medical knowledge, but also an emotional exchange. Patients’ distress decreases when their clinicians were empathic and generated trust^27,32^.

The regression results showed that age is also a factor affecting distress. Compared with people younger than 50 years old, patients aged 50–60 years have a higher risk of distress. This may be because the age of 50–60 is still the backbone of the society and the family in the current Chinese society, bearing the heavy burden of the society and the family. When a certain disease is diagnosed, the negative emotions caused by it are heavier. Bivariable analysis by Freiman et al. showed that age over 65 years was a protective factor for distress in patients with pulmonary nodules^33^. Similarly, the results of Li et al. showed that patients with pulmonary nodules between the ages of 40 and 60 had the highest proportion of anxiety, but it was not statistically significant^34^. Further research is needed on the association of age with distress in patients with pulmonary nodules.

We found that anxiety about the next test result, but not worry about lung cancer, was an influencing factor of distress. Similar to breast cancer screening, when an individual has a false-positive screening result, it may experience a period of distress. The difference is that distress in false-positive individuals of breast cancer screening is transient, and it disappears immediately after a negative biopsy result^26^. Many patients with pulmonary nodules may be required to follow regular surveillance, leaving them in a state of uncertainty for months or even years. Consequently, the next test results were more closely and directly related to their mood compared with cancer.

### Limitations

We should mention that this study has several limitations. Since the design is a cross-sectional study, there may be attentional bias and selection bias in the study. The researchers investigated outpatient patients with pulmonary nodules in a tertiary hospital of a provincial capital city. The level of medical care varies among hospitals of different levels, which may affect the distress among patients with pulmonary nodules. Due to differences in the description of pulmonary nodules in imaging reports among different physicians, the characteristics of individual pulmonary nodules were not included in this study. In addition, the quality of doctor-patient communication is measured through patient self-assessment. To truly reflect the quality of communication, it is beneficial to comprehensively consider the evaluation of communication quality by doctors and third parties. Due to the limited sample size, the odds ratio of age group 50–60 might inflated^35^. Finally, Due to the limitations of the cross-sectional study, the causal relationship between the variables needs to be further investigated.

## Conclusion

We found that distress levels were moderate in patients with pulmonary nodules. Age, higher decision conflicts scores, lower physician–patient communication quality score, and being anxious about the results of future tests or treatments are influencing factors of distress among pulmonary nodule patients.

### Practice implications

In the screening process, in addition to the patients who are finally diagnosed with lung cancer, patients with positive results after screening also need more attention. Due to the heavy clinical tasks of doctors, nurses may play an important role in the health guidance of patients with pulmonary nodules. In this process, patients are informed of the alternative pulmonary nodules management options and the pros and cons of each option. Patient's own decision-making preferences needs more attention, and therapeutic alliance needs to be established between doctor, nurse and patients. This patient-centered communication method can not only effectively eliminate the decision conflicts, but also allow patients to participate in decision-making, which greatly improves the quality of communication and decision. Of course, the feasibility and effectiveness of this model need further experiments to confirm.

## Data Availability

The data that support the findings of this study are available on request from the corresponding author.
